# Supplementary material for: Molecular imaging of liver inflammation using an anti-VCAM-1 nanobody
Source: Nat Commun. 2023 Feb 24;14:1062. doi: 10.1038/s41467-023-36776-7 (PMC9957989; doi:10.1038/s41467-023-36776-7)
Supplement: Supplementary file 1 — Supplementary Information [file 41467_2023_36776_MOESM1_ESM.pdf]

Supplementary information

Molecular imaging of liver inflammation using an anti-VCAM-1 nanobody

Maxime Nachit, Christopher Montemagno et al.

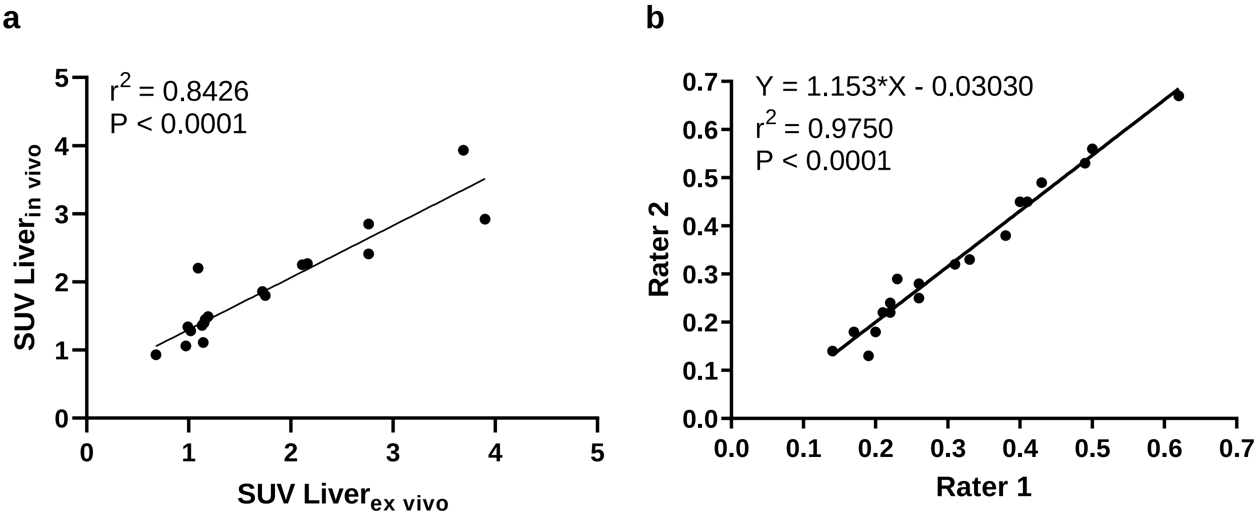

Intraclass correlation coefficient

Number of subjects (n)	19
Number of raters (k)	2
Model	The same raters for all subjects.<br>Two-way model.
Type	Absolute agreement
Measurements	Rater_1<br>Rater_2

Intraclass Correlation Coefficient

	Intraclass correlation [a]	95% Confidence Interval
Single measures [b]	0,9694	0,9029 to 0,9890
Average measures [c]	0,9845	0,9490 to 0,9945

[a] The degree of absolute agreement among measurements.
[b] Estimates the reliability of single ratings.
[c] Estimates the reliability of averages of *k* ratings.

Supplementary Figure 1. Proof-of-concept study for ^{99m}Tc-cAbVCAM1-5 liver imaging (n=10 V-STD and n=9 V-MCD). **a** Correlation between ^{99m}Tc-cAbVCAM1-5 uptake expressed in SUV obtained in vivo by SPECT imaging and ex vivo by liver sampling (p<0.0001). **b** Intra-class correlation coefficients (ICC) were calculated using two-way random and absolute agreement (Medcalc 12.7) to evaluate the reproducibility of the SPECT liver quantification method between 2 distinct raters (CM and RC). 95% confidence interval on ICC (0.94) is given in the table below the correlation obtained with a p value <0.0001. SUV: Standardized Uptake Value, STD: standard diet, MCD: Methionine and Choline deficient diet, V: ^{99m}Tc-cAbVCAM1-5 nanobody. Source data are provided as a Source Data file.

Biodistribution (SUV)	V-STD-diet	C-MCD-diet	V-MCD-diet
Liver	0.26 ± 0.03*	0.18 ± 0.03	0.41 ± 0.14*#
Blood	0.11 ± 0.02*	0.17 ± 0.04	0.26 ± 0.13#
Heart	0.07 ± 0.01	0.07 ± 0.02	0.11 ± 0.04
Muscle	0.02 ± 0.00	0.03 ± 0.01	0.03 ± 0.02
Spleen	2.01 ± 0.36*	0.11 ± 0.03	4.41 ± 2.11*#
Lymph nodes	0.96 ± 0.43*	0.12 ± 0.05	1.30 ± 0.65*
Bone marrow	1.08 ± 0.44*	0.03 ± 0.01	1.18 ± 0.50*
Stomach	0.12 ± 0.01	0.11 ± 0.03	0.17 ± 0.04
Kidneys	64.51 ± 7.08	69.94 ± 20.15	54.65 ± 10.47

Supplementary Table 1. ^{99m}Tc-cAbVCAM1-5 biodistribution by ex vivo gamma-well counting of organs from mice after 8 weeks of diet. Data are expressed as SUV: organ activity (MBq/g of organ) / injected dose (MBq / BW (g)), and presented as mean ± standard deviation. C-MCD & V-STD mice (n=10) and V-MCD mice (n=9). Mann and Whitney test for unpaired values: * *P*<0.01 vs C-MCD, # *P*<0.01 vs V-STD. Source data are provided as a Source Data file.

Manuscript part	Genetic background	Genotype	Diet	Abbreviation	Number of animals	NAFLD	Steatohepatitis	Metabolic syndrome
**Proof-of-concept**	C57Bl6/j	Wild-type	Methionine-choline deficient	MCD	20*	-	Severe	Absent
	C57Bl6/j	Wild-type	Standard chow	STD	10	-	Absent	Absent
**Main study**	NOD.B10	Wild-type	Standard chow	Ctl	8	Absent	Absent	Absent
	NOD.B10	Wild-type	High fat	WH	6	Mild	Mild	Mild
	NOD.B10	FOZ	High fat	FH	6	Severe	Severe	Severe
	C57Bl6/j	Wild-type	Choline-supplemente d high fat	CSH	6	Mild	Mild	Mild
	C57Bl6/j	Wild-type	Choline-deficient high fat diet	CDH	6	Severe	Severe	Absent

Supplementary Table 2. Preclinical models characteristics.*one mouse was excluded in V-MCD group since a limit point was reached at W8 with MCD diet (loss of BW > 40%).

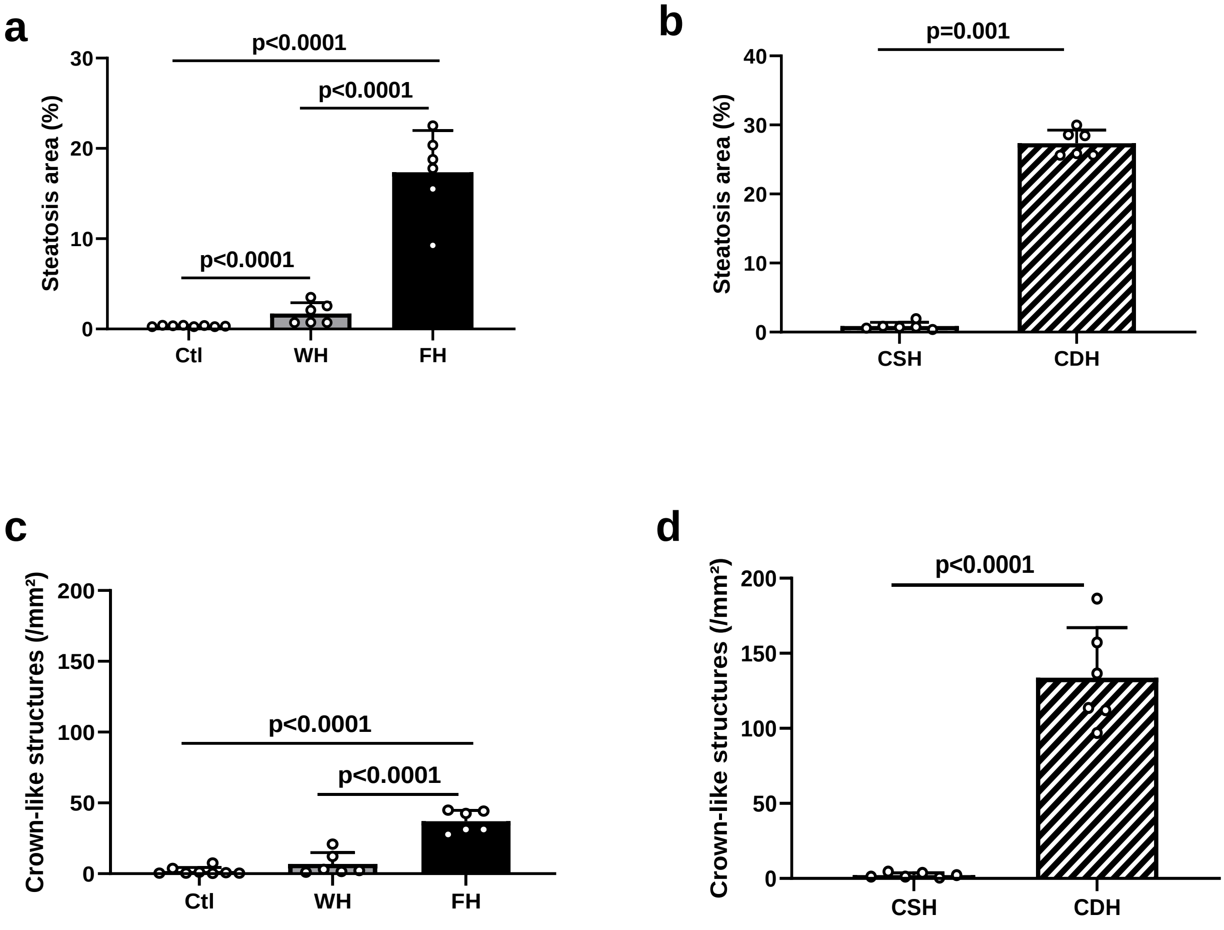

Supplementary Figure 2. Main study: histological analysis. **a** Steatosis area evaluated on liver slides from WT mice fed a control diet (Ctl, n=8) or high fat diet (WH, n=6), and Foz mice fed a high fat diet (FH, n=6). **b** Steatosis area evaluated on liver slides from mice fed a high fat diet plus a choline-supplemented diet (CSH, n=6) or a deficient-choline diet (CDH, n=6). **c** Number of Crown-like structures per mm^2 evaluated on liver slides from WT mice fed a control diet (Ctl, n=8) or high fat diet (WH, n=6), and Foz mice fed a high fat diet (FH, n=6). **d** Number of Crown-like structures per mm^2 evaluated on liver slides from mice fed a high fat diet plus a choline-supplemented diet (CSH, n=6) or a deficient-choline diet (CDH, n=6). All values are presented as mean ± SD. Mann and Whitney test for unpaired values, p values are given above bars. Source data are provided as a Source Data file.

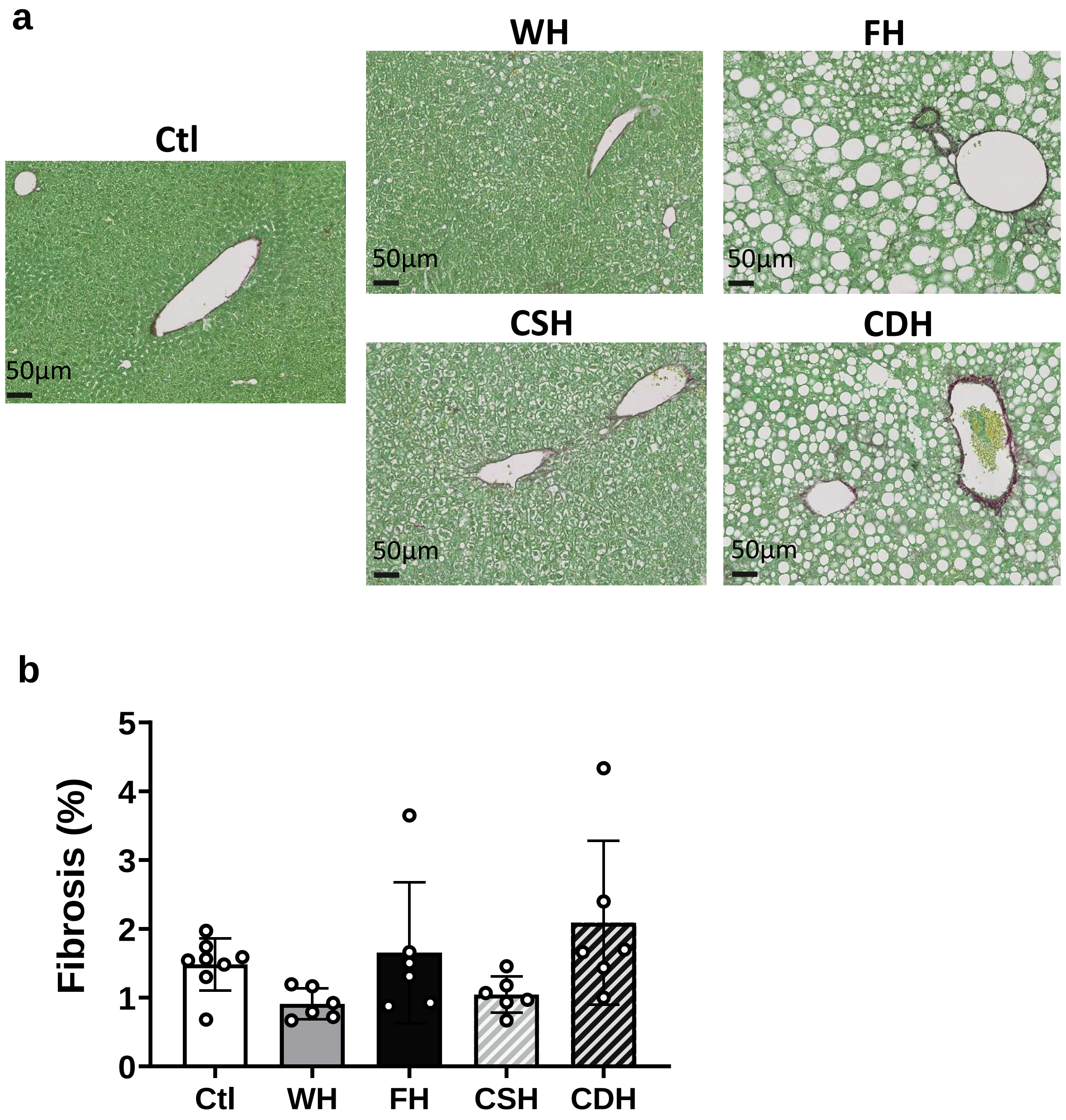

Supplementary Figure 3. Sirius red staining of liver sections in WT mice fed a normal diet (Ctl, n=8) or a high fat diet (WH, n=6), in FOZ mice fed a high fat diet (FH, n=6) and in C57BL6/J mice fed a fat-rich choline supplemented (CSH, n=6) or deficient (CDH, n=6) diet. **a** Representative histological picture. **b** Morphometrical quantification using QuPath v0.3.0. Data expressed as mean ± SD; comparison using one-way ANOVA, corrected for multiple comparisons using Tukey test, 95% confidence interval (not significant when $p>0.05$). Source data are provided as a Source Data file.

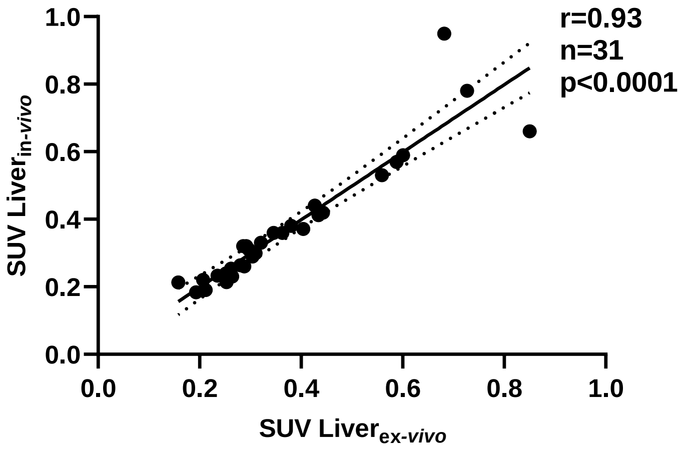

Supplementary Figure 4. Correlation between ^{99m}Tc-cAbVCAM1-5 uptake expressed in SUV obtained in vivo by SPECT imaging and ex vivo by liver sampling. Source data are provided as a Source Data file.

Biodistribution (SUV)	Ctl	WH	FH
Liver	0.22 ± 0.04	0.32 ± 0.08*	0.35 ± 0.07*
Blood	0.15 ± 0.04	0.24 ± 0.12	0.27 ± 0.07*
Heart	0.10 ± 0.02	0.14 ± 0.05	0.17 ± 0.04*
Muscle	0.04 ± 0.02	0.05 ± 0.03	0.05 ± 0.01
Spleen	2.53 ± 0.44	3.86 ± 1.25*	3.92 ± 0.46*
Lymph nodes	0.76 ± 0.22	0.94 ± 0.32	0.92 ± 0.42
Bone marrow	2.19 ± 0.36	2.57 ± 1.33	2.84 ± 0.28
Stomach	0.14 ± 0.02	0.24 ± 0.08*	0.29 ± 0.02*
Kidneys	48.23 ± 3.26	52.25 ± 11.40	52.69 ± 5.68

Supplementary Table 3. ^{99m}Tc-cAbVCAM1-5 biodistribution by ex vivo gamma-well counting of organs from mice after 12 weeks of diet. Data are expressed as SUV: organ activity (MBq/g of organ) / injected dose (MBq / BW (g)) and presented as mean ± standard deviation. n=8 (Ctl) or n=6 (WH/FH) / group, Mann and Whitney test for unpaired values: * *P*<0.05 vs Ctl, # *P*<0.05 vs WH, non significant when p>0.05. Source data are provided as a Source Data file.

Biodistribution (SUV)	CSH	CDH
Liver	0.32 ± 0.03	0.67 ± 0.11*
Blood	0.21 ± 0.03	0.26 ± 0.05
Heart	0.12 ± 0.02	0.15 ± 0.03
Muscle	0.05 ± 0.01	0.04 ± 0.01
Spleen	2.47 ± 0.38	2.90 ± 0.53
Lymph nodes	1.39 ± 0.59	1.20 ± 0.57
Bone marrow	2.70 ± 1.03	2.60 ± 0.52
Stomach	0.23 ± 0.04	0.23 ± 0.05
Kidneys	64.21 ± 4.93	58.91 ± 5.38

Supplementary Table 4. ^{99m}Tc-cAbVCAM1-5 biodistribution by ex vivo gamma-well counting of organs from mice after 4 weeks of diet. Data are expressed as SUV: organ activity (MBq/g of organ) / injected dose (MBq / BW (g)) and presented as mean ± standard deviation. n= 6 / group, Mann and Whitney test for unpaired values: * *P*<0.0001 vs CSH, non significant when p>0.05. Source data are provided as a Source Data file.

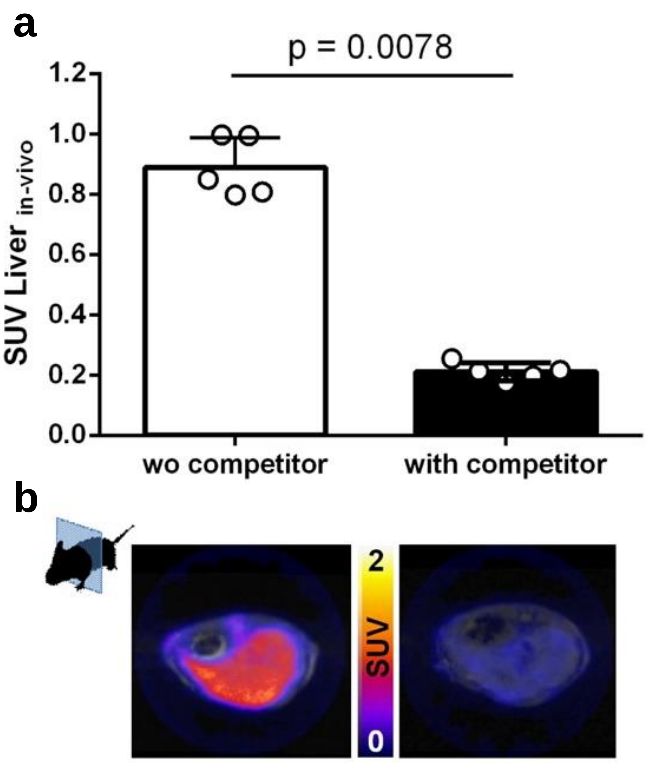

Supplementary Figure 5. In vivo competition study. ^{99m}Tc-cAbVCAM1-5 was injected in CDH-fed mice either alone (n=5) or together with a 100-fold excess of unlabeled competitor cAbVCAM1-5 (n=5). **a** Quantification of liver SPECT imaging (Data expressed as mean ± SD); **b** Representative liver SPECT/CT images. Competition resulted in a significant decrease of ^{99m}Tc-cAbVCAM1-5 uptake in liver (~76.4%), thereby demonstrating specificity of the signal. Mann and Whitney test for unpaired values, p value given above the bars. Source data are provided as a Source Data file.

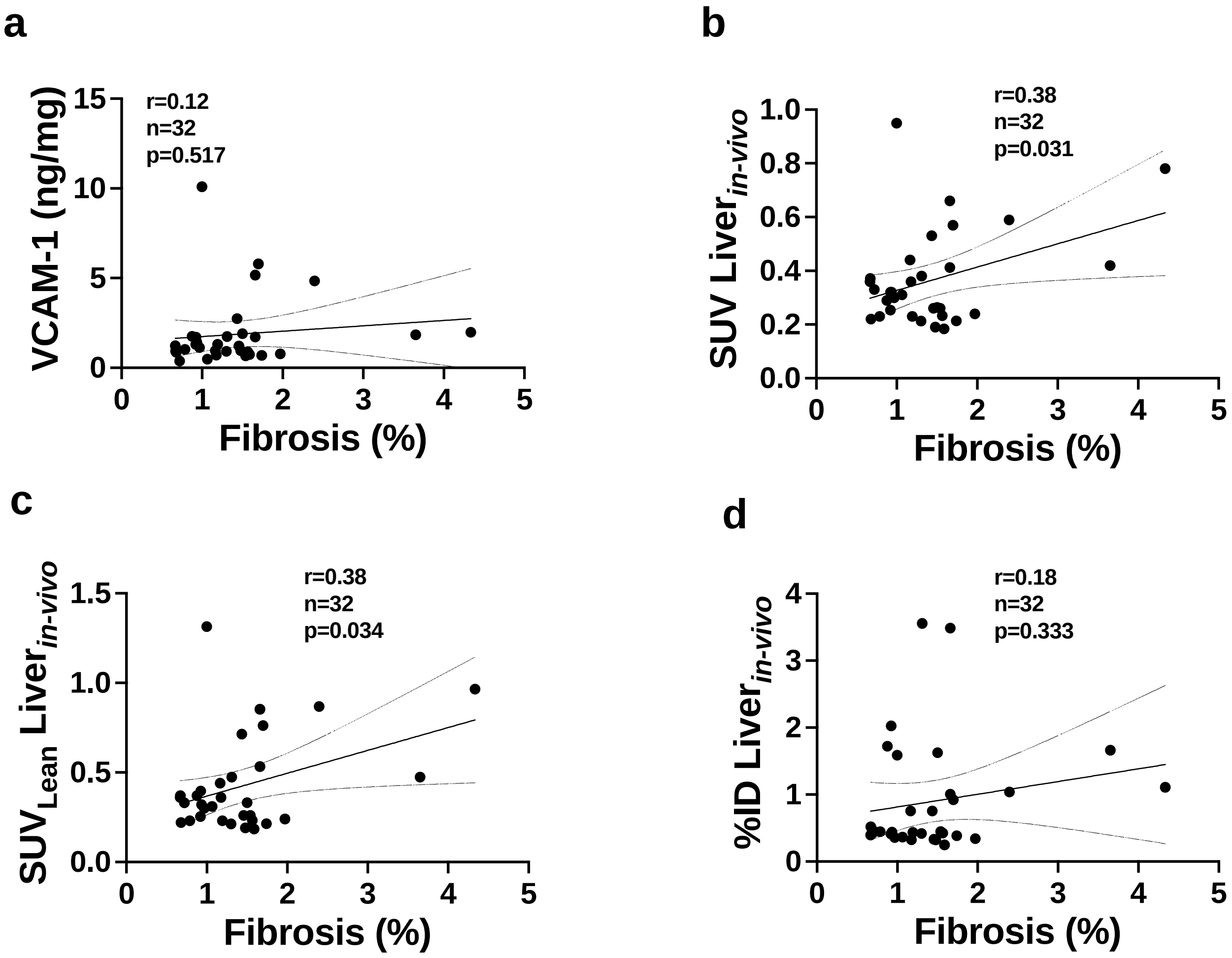

Supplementary Figure 6. Pearson's correlation between the histologically-determined percentage area of liver fibrosis and **a** VCAM-1 protein liver content, **b** SUV Liver$_{in\text{-}vivo}$ **c** SUV$_{Lean}$ Liver$_{in\text{-}vivo}$ and **d** Total liver uptake (%ID) of ^{99m}Tc-cAbVCAM1-5. Source data are provided as a Source Data file.

	SUV			SUV_Lean			%ID		
Characteristic	Beta	95% CI[1]	p-value	Beta	95% CI[1]	p-value	Beta	95% CI[1]	p-value
Ballooning									
0	—	—		—	—		—	—	
1	0.13	-0.06, 0.33	0.2	0.14	-0.14, 0.41	0.3	0.39	-0.45, 1.2	0.3
2	-0.25	-0.36, -0.15	<0.001	-0.35	-0.50, -0.21	<0.001	1.5	1.0, 1.9	<0.001
Inflammation									
0	—	—		—	—		—	—	
1	0.07	-0.02, 0.17	0.11	0.07	-0.06, 0.20	0.3	-0.05	-0.44, 0.35	0.8
2	0.26	0.12, 0.40	<0.001	0.37	0.17, 0.56	<0.001	-0.03	-0.62, 0.57	>0.9
3	0.41	0.32, 0.51	<0.001	0.63	0.50, 0.77	<0.001	0.81	0.40, 1.2	<0.001
Fibrosis	0.02	-0.03, 0.07	0.5	0.01	-0.06, 0.08	0.8	-0.15	-0.36, 0.06	0.2

[1] CI = Confidence Interval

Fibrosis is expressed in % of stained area

Supplementary Table 5. Linear regression to identify histological predictor of VCAM-1 signal quantification (SUV, SUV_{Lean} or %ID of the liver in vivo). Inflammation and ballooning, but not fibrosis, are histological predictors of VCAM-1 signal (n=32).

SUV Liver$_{in\text{-}vivo}$

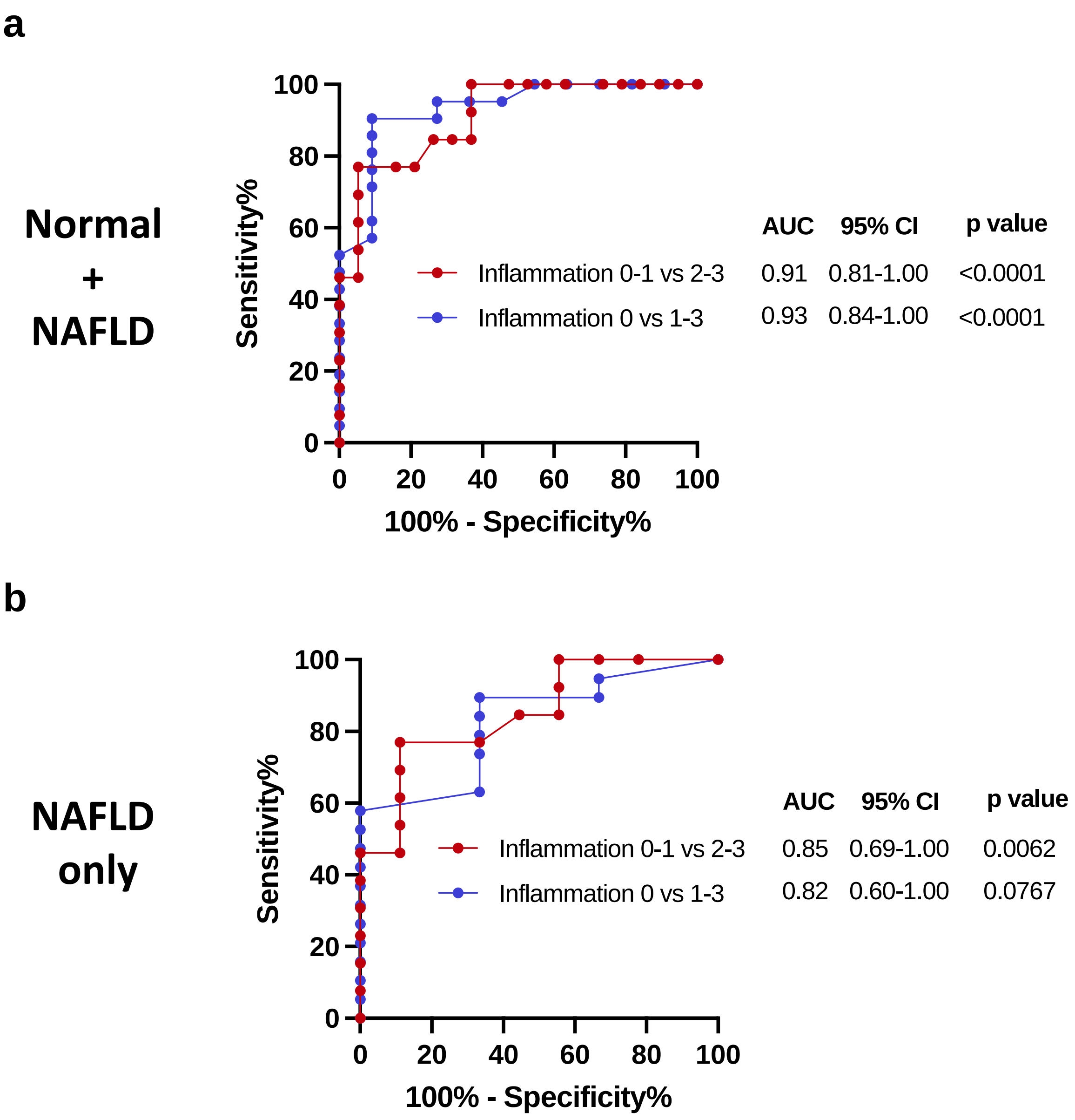

Supplementary Figure 7. ROC curves for detection of liver inflammation in **a** the entire cohort (Normal + NAFLD) or **b** only in animals with liver steatosis (NAFLD only). AUC: area under the curve calculated using Wilson/Brown method, 95% confidence interval (CI) and p values are provided on each panel. Source data are provided as a Source Data file.

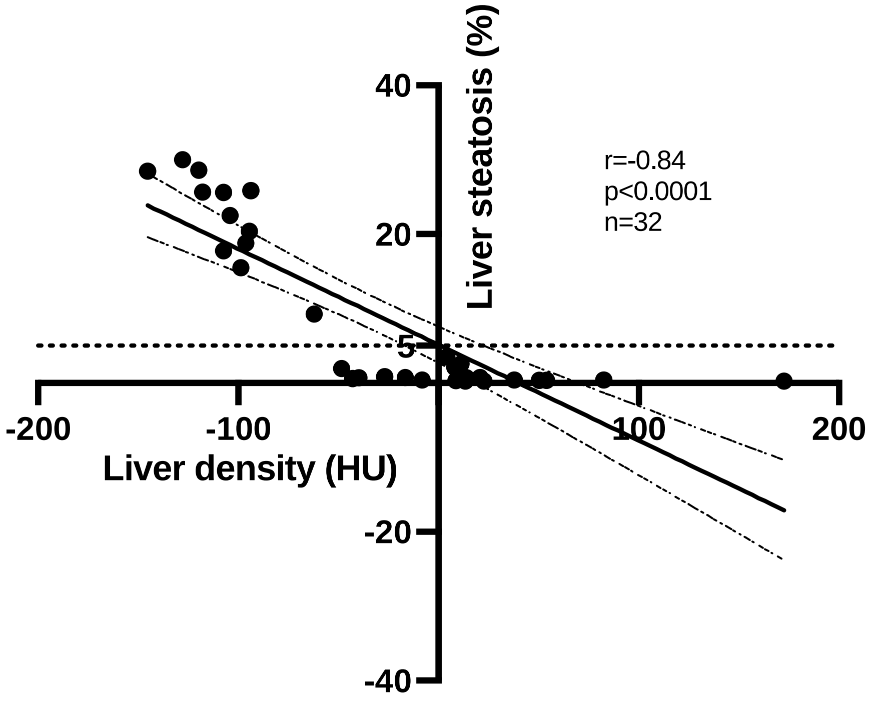

Supplementary Figure 8. Pearson's correlation between liver density in Hounsfield Unit (HU) and histological steatosis in %. Source data are provided as a Source Data file.

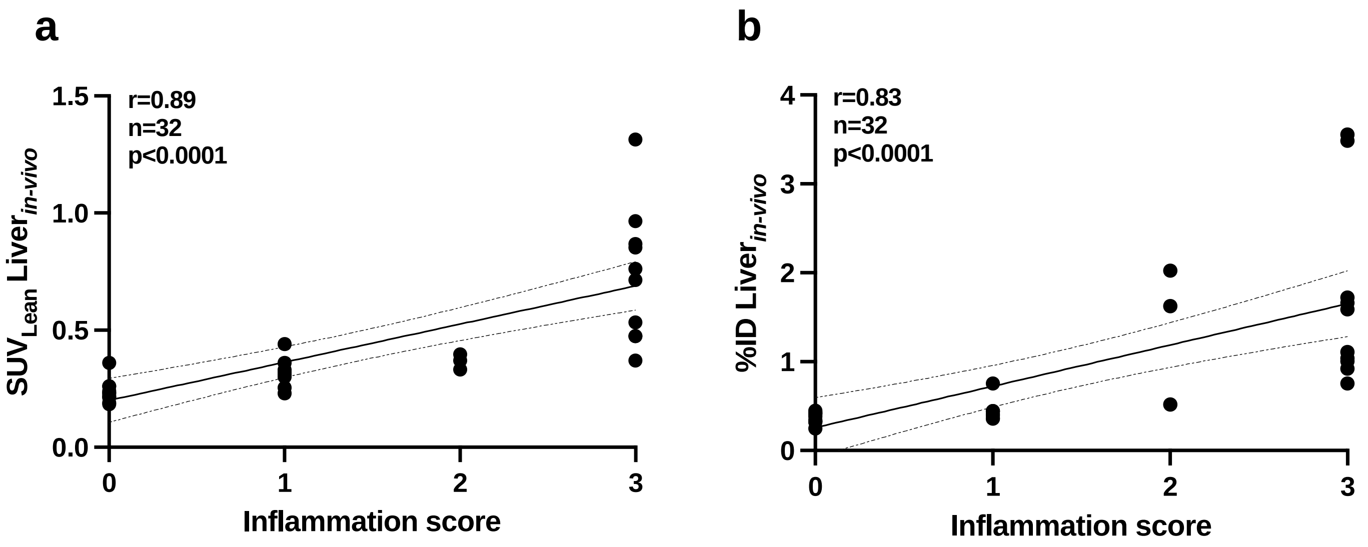

Supplementary Figure 9. Spearman's correlation between histological inflammatory score and **a** SUV_{Lean} Liver$_{in\text{-}vivo}$ and **b** %ID Liver$_{in\text{-}vivo.}$ Source data are provided as a Source Data file.

Actin - Forward	5’-CTCCTGAGCGCAAGTACTCC-3’
Actin - Reverse	5’-TGTTTTCTGCGCAAGTTAGG-3 ’
VCAM-1 - Forward	5’-GCCACCCTCACCTTAATTGC-3’
VCAM-1 - Reverse	5’-TCAGAACAACCGAATCCCCA-3’
MCP-1 - Forward	5’-TGATCCCAATGAGTAGGCTGGAG-3’
MCP-1 - Reverse	5’-ATGTCTGGACCCATTCCTTCTTG-3’
EMR1 (F4:80) - Forward	5’- TGACTCACCTTGTGGTCCTAA-3’
EMR1 (F4:80) - Reverse	5’ -CTTCCCAGAATCCAGTCTTTCC-3’

Supplementary Table 6. Primer sequences employed for the qPCR.
