# Supplementary material for: Molecular imaging of liver inflammation using an anti-VCAM-1 nanobody
Source: Nat Commun. 2023 Feb 24;14:1062. doi: 10.1038/s41467-023-36776-7 (PMC9957989; doi:10.1038/s41467-023-36776-7)
Supplement: Supplementary file 3 — Reporting Summary [file 41467_2023_36776_MOESM3_ESM.pdf]

nature portfolio

Corresponding author(s): Alexis Broisat

Last updated by author(s): 25.01.2023

Reporting Summary

Nature Portfolio wishes to improve the reproducibility of the work that we publish. This form provides structure for consistency and transparency in reporting. For further information on Nature Portfolio policies, see our Editorial Policies and the Editorial Policy Checklist.

Statistics

For all statistical analyses, confirm that the following items are present in the figure legend, table legend, main text, or Methods section.

n/a	Confirmed	
☐	☒	The exact sample size (*n*) for each experimental group/condition, given as a discrete number and unit of measurement
☐	☒	A statement on whether measurements were taken from distinct samples or whether the same sample was measured repeatedly
☐	☒	The statistical test(s) used AND whether they are one- or two-sided<br>*Only common tests should be described solely by name; describe more complex techniques in the Methods section.*
☐	☒	A description of all covariates tested
☐	☒	A description of any assumptions or corrections, such as tests of normality and adjustment for multiple comparisons
☐	☒	A full description of the statistical parameters including central tendency (e.g. means) or other basic estimates (e.g. regression coefficient) AND variation (e.g. standard deviation) or associated estimates of uncertainty (e.g. confidence intervals)
☐	☒	For null hypothesis testing, the test statistic (e.g. *F*, *t*, *r*) with confidence intervals, effect sizes, degrees of freedom and *P* value noted<br>*Give P values as exact values whenever suitable.*
☒	☐	For Bayesian analysis, information on the choice of priors and Markov chain Monte Carlo settings
☒	☐	For hierarchical and complex designs, identification of the appropriate level for tests and full reporting of outcomes
☐	☒	Estimates of effect sizes (e.g. Cohen's *d*, Pearson's *r*), indicating how they were calculated

Our web collection on statistics for biologists contains articles on many of the points above.

Software and code

Policy information about availability of computer code

Data collection	Vivoquant v4.0 (Invicro) was used to extract data from SPECT images.
Data analysis	Most analysis were conducted with GraphPad 9 (Prism), except multivariate analysis (R v4.2.1) and intra-class correlation coefficients (ICC) calculated using two-way random and absolute agreement (Medcalc 12.7). Liver fibrosis was quantified with QuPath v0.3.0 (opensource).

For manuscripts utilizing custom algorithms or software that are central to the research but not yet described in published literature, software must be made available to editors and reviewers. We strongly encourage code deposition in a community repository (e.g. GitHub). See the Nature Portfolio guidelines for submitting code & software for further information.

Data

Policy information about availability of data

All manuscripts must include a data availability statement. This statement should provide the following information, where applicable:

- Accession codes, unique identifiers, or web links for publicly available datasets
- A description of any restrictions on data availability
- For clinical datasets or third party data, please ensure that the statement adheres to our policy

All data presented in graphs within the Figures & Supplementary Figures are provided in the Source Data file. All images (IHC and SPECT/CT) are available from the corresponding author [AB] upon reasonable request. Source data are provided with this paper.

Human research participants

Policy information about studies involving human research participants and Sex and Gender in Research.

Reporting on sex and gender	NA
Population characteristics	NA
Recruitment	NA
Ethics oversight	NA

Note that full information on the approval of the study protocol must also be provided in the manuscript.

Field-specific reporting

Please select the one below that is the best fit for your research. If you are not sure, read the appropriate sections before making your selection.

- [x] Life sciences
- [ ] Behavioural & social sciences
- [ ] Ecological, evolutionary & environmental sciences

For a reference copy of the document with all sections, see nature.com/documents/nr-reporting-summary-flat.pdf

Life sciences study design

All studies must disclose on these points even when the disclosure is negative.

Sample size	The proof-of-concept study demonstrated that 99mTc-cAbVCAM1-5 uptake was up to 80% higher in a prototypical model of steatohepatitis (MCD) vs control mice (STD). We posited that the difference in 99mTc-cAbVCAM1-5 uptake might be less marked (and the variability higher) in clinically-relevant mouse models. Hence, for an estimated difference of 50% in 99mTc-cAbVCAM1-5 uptake and a standard deviation at least two-fold higher than what was observed in STD mice, we estimated a minimal n= 6 animals in each groups to reach an alpha = 0.05% and a Power = 80%.
Data exclusions	One mouse from the V-MCD group was excluded from the analyses because she reached a limit point of the ethical protocol (more than 40% loss of body weight)
Replication	A minimum of n=6 mice per group were used. All attempts at replication were successful.
Randomization	In the competition experiments, mice were randomly allocated to receive an irrelevant nanobody (proof-of-concept) or a cold cAbVCAM1-5 (main study).
Blinding	Given the large difference of phenotypes (e.g. BW) in different mice models, blinding was not realistic. However, we quantified 99mTc-cAbVCAM1-5 in the entire liver for each animals, leaving little room for possible sampling bias. Likewise, most data analyses were performed with all group combined (e.g. ROC curves).

Reporting for specific materials, systems and methods

We require information from authors about some types of materials, experimental systems and methods used in many studies. Here, indicate whether each material, system or method listed is relevant to your study. If you are not sure if a list item applies to your research, read the appropriate section before selecting a response.

Materials & experimental systems

n/a	Involved in the study	
[ ]	[x]	Antibodies
[x]	[ ]	Eukaryotic cell lines
[x]	[ ]	Palaeontology and archaeology
[ ]	[x]	Animals and other organisms
[x]	[ ]	Clinical data
[x]	[ ]	Dual use research of concern

Methods

n/a	Involved in the study	
[x]	[ ]	ChIP-seq
[x]	[ ]	Flow cytometry
[x]	[ ]	MRI-based neuroimaging

Antibodies

Antibodies used	Two nanobodies were used in imaging experiments (cAbVCAM1-5 and cAbBcII10) and they were both produced by the authors, not commercialized, available on request if necessary.<br>Commercialized antibodies : rat anti-mouse Mac-2 antibody (Ref catalog CL8942AP, CedarlaneLabs, ref 1 as exemple of validation), rat anti-mouse F4/80 antibody (MCA497G, BioRad, ref 2&3 as examples of validation) for IHC; and anti-VCAM-1 protein for Elisa (Abcam, SimpleStep ELISA ab201278, validation on the website of Abcam).
Validation	The two main nanobodies produced by the authors have been previously published and validated : Broisat A et al Circ Res 2012, 110 (7):927-37 and Broisat et al J Nucl Med. 2014, 55(10):1678-84, as most relevant examples.<br>Ref 1. Kanter JE, et al. (2012). PNAS. 109(12): E715-E724; Ref 2. Gordon, S. et al. (1992) Curr Top Microbiol Immunol. 181: 1-37 & Ref 3. Warschkau, H. & Kiderlen, A.F. (1999) J Immunol 163(6):3409-16.<br>https://www.abcam.com/mouse-vcam1-elisa-kit-cd106-ab201278.html for SimpleStep ELISA ab201278

Animals and other research organisms

Policy information about studies involving animals; ARRIVE guidelines recommended for reporting animal research, and Sex and Gender in Research

Laboratory animals	Two strains were used: 12 weeks-old male C57BL6/J mice (STD, MCD, CDH and CSH) and 12 weeks-old male NOD.B10 mice (WT, WH, FH). Housing conditions have been added in the manuscript: Mice were on a 12-h light/dark cycle, temperature was maintained between 20 and 24°C and a relative humidity of 40-60%.
Wild animals	No wild animal were used in this study
Reporting on sex	Only male animals were used as they were extensively used as reproducible model of non-alcoholic fatty liver disease (NAFLD). Human data support no difference in liver VCAM-1 expression between biological male and female (DOI: 10.1172/JCI147556) and follow-up experiments in female animals are planned.
Field-collected samples	No field-collected samples were used in this study
Ethics oversight	All procedures were approved by the animal care and ethic committee of Grenoble Alpes University (Cometh', protocols ref: APAFIS#2993-2015120219565475 & APAFIS#23780-2020012412159346_v2) and the ad hoc French minister as well as by Belgian institutions (protocol ref: 2016/UCL/MD/003). A one-week acclimatization period was respected prior diet onset and, when applicable, randomization was performed so that mean weights were similar between groups.

Note that full information on the approval of the study protocol must also be provided in the manuscript.
